# Novel coronavirus (COVID-19) infection: What a doctor on the frontline needs to know

**DOI:** 10.1016/j.amsu.2020.05.014

**Published:** 2020-05-13

**Authors:** Billy Down, Sagar Kulkarni, Ameer Hamid Ahmed Khan, Benjamin Barker, Ivan Tang

**Affiliations:** a Milton Keynes University Hospital NHS Foundation Trust, Standing Way, Milton Keynes, Buckinghamshire, MK6 5BY, United Kingdom; b Oxford University Clinical Academic Graduate School, Medical Sciences Division, University of Oxford, John Radcliffe Hospital, Headington, Oxfordshire, OX3 9DU, United Kingdom; c Barking, Havering and Redbridge University Hospitals NHS Trust, Rom Valley Way, Romford, RM7 0AG, United Kingdom

**Keywords:** Coronavirus, COVID-19, Junior doctor, Pandemic, Pneumonia

## Abstract

Coronavirus disease 2019 (COVID-19) is a zoonotic respiratory infection originating from Wuhan, China. Rapidly spreading from Wuhan to all inhabited continents of the world, the World Health Organisation declared COVID-19 a pandemic on March 11, 2019. Infected patients present with fever and cough; radiological features include bilateral infiltrates on chest x-ray and computed tomography scanning. Management is supportive with oxygen supplementation, broad-spectrum antibiotics as well as careful fluid balancing. A number of drugs, both new and old, are currently in clinical trials and being used on an experimental basis in clinical practice. The COVID-19 pandemic is the greatest worldwide public health crisis of a generation, and has led to seismic political, economic and social changes. This review provides an overview of COVID-19 for junior doctors who find themselves on a new frontline of healthcare.

## Highlights

• Coronavirus disease 2019 is a zoonotic respiratory infection that rapidly spread from China to become a pandemic.
• The most common symptoms are cough, fever and shortness of breath; infection typically produces mild symptoms.
• A minority of patients have severe symptoms, such as respiratory failure, which requires hospital admission and even intensive care unit admission.
• Guidelines suggest that the management of coronavirus disease 2019, in most cases, is supportive
• Several agents, such as hydroxychloroquine, azithromycin and remdesivir are currently under investigation as treatment for the disease.

## 1 Introduction

### 1.1 Origins of COVID-19

The COVID-19 outbreak can be traced to an unusual cluster of 27 pneumonia cases with unknown aetiology in Wuhan, China (1). The Hunan Seafood Wholesale Market which hosted a variety of exotic live and deceased wildlife including bamboo rats, badgers and wolf clubs was a common reported link. The Wuhan Municipal Health Commission subsequently reported this case cluster to the World Health Organization (WHO) on the 31st December 2019. Reports of the first fatality came on the 1^st^ January 2020 and the Hunan Seafood Wholesale Market was closed. On the 2nd January, the virus was identified as the 2019-novel Coronavirus and its genetic sequence shared with the WHO [2]. By the 7th January, the 2019-novel Coronavirus had already been isolated by the Chinese Center for Disease Control. The WHO subsequently renamed the 2019-novel Coronavirus as Severe Acute Respiratory Syndrome Coronavirus 2 (SARS-CoV-2) and the disease resulting from the referred to as Coronavirus Disease 2019 (COVID-19).

### 1.2 Global spread of Covid-19

Wuhan is the capital of central China's Hubei province and is itself is a major hub of economic activity. It has a population of 11 million. Every day, 3500 people fly internationally from the province (1). Within 30 days of outbreak reports, COVID-19 had already spread from Wuhan to other Chinese settlements and the number of confirmed cases rose to 9000 (more than the number of SARS and MERS cases reported to date) [3].

Multiple domestic measures to contain the outbreak were enforced including the banning of wildlife and poultry in wet markets, contact tracing of cases, increasing lockdown and quarantine restrictions, school closures and prohibition of large gatherings. Similarly, neighbouring countries commenced airport screening, travel bans and quarantining of evacuated citizens from the Hubei province [1]. The first reported case of COVID-19 was confirmed in Thailand on the 13th January and was followed by a case in Japan by the 15th and South Korea by the 20th. Despite ongoing efforts, the number of Chinese cases continued to rise and evidence emerged of growing clusters of cases abroad indicating human-human transmission outside China.

The WHO issued a statement on the 19th January warning of human-human transmission and by the 31st it was announced that COVID-19 was now a Public Health Emergency of International Concern and calls were made for an international coordinated response [4]. By the 11th March, COVID-19 was declared a pandemic [2].

### 1.3 Pathophysiology

The SARS-CoV-2 virus belongs to the family of coronaviruses which are enveloped, positive-stranded RNA viruses with nucleocapsids [1]. Six other human coronaviruses have been identified to date, including SARS-CoV, responsible for the SARS outbreak of 2003, and MERS-CoV, responsible for the Middle East Respiratory Syndrome (MERS) outbreaks of 2015 and 2018 [3]. SARS-CoV2 shares a 96.2% genetic sequence homology with a bat coronavirus and it is currently thought that it was introduced to humans via an unknown intermediate animal vector [1] before progressing to propagation via human-to-human transmission [2].

#### 1.3.1 Routes of transmission

Viral transmission of SARS-CoV-2 occurs directly via respiratory droplets when infected individuals cough, sneeze or talk in close proximity to others and indirectly via fomites objects or surfaces. Although some studies have suggested SARS-CoV2 virus may remain viable for at least 3 h in experimentally generated aerosols, it is not fully clear on whether this has clinical implications in the epidemiology of COVID-19 [5].

#### 1.3.2 Infectivity

Incubation times have been estimated to range between 1 and 14 days (median duration of 5 days) whilst the median duration of viral shedding is around 20 days in those who recover [[6], [7], [8]]. The reproductive number of SARS-CoV-2 (referring to the number of people acquiring the infection from one infected person) is around 2.68 (95% confidence interval: 2.47–2.86) [6,[9], [10], [11]].

#### 1.3.3 Pathogenesis

The pathogenesis of SARS-CoV2 remains poorly understood and still requires further elucidation. Research on other viruses from the coronavirus family such as SARS-CoV and MERS-CoV may help to provide analogous mechanistic insights.

One notable structural feature of coronaviruses is that they have spike glycoproteins. In the case of SARS-Cov-2, receptor-binding domains on these spike glycoproteins are responsible for viral entry into host cells via angiotensin-converting-enzyme-2 (ACE2) receptors. These spike glycoprotein receptor-binding domains in the SARS-CoV-2 virus appear to have a greater affinity to ACE-2 receptors than the SARS-CoV virus and given the high conservation of the S2 subunit, these receptor-binding domains have been identified as possible targets for antiviral compounds [12]. ACE2 receptors have higher expression in the lungs, heart, oesophagus, kidneys, bladder and ileum and thus these organs are considered more vulnerable to SARS-CoV2 infection [6].

#### 1.3.4 Host immune responses

The host immune response to SARS-CoV-2 is still not fully understood. Viral antigens are likely presented to host immune cells to trigger humoral and cellular immunity by virus-specific B and T lymphocytes. SARS-CoV2 is likely to trigger IgM and IgG production in a similar humoral immunity response to SARS-CoV [13]. Further research may be useful in understanding immunity in patients who have had COVID-19 and also the value of potential screening antibody tests.

#### 1.3.5 Complications

Similar to the SARS-CoV and MERS-CoV viruses, the SARS-CoV-2 virus can trigger a violent and potentially fatal dysregulated immune host response. This response is thought to be mediated by the triggered release of large numbers of pro-inflammatory cytokines and chemokines in a ‘cytokine storm’ by immune effector cells and can result in Acute Respiratory Distress Syndrome (ARDS) and multiple organ failure [13]. ARDS is a common immunopathological event for SARS-CoV-2 and has been identified early on as the main cause of death in patients with COVID-19 [14].

## 2 Clinical presentation and assessment

The clinical history is critical to determine the risk of COVID-19. Symptoms of the disorder resemble those of a viral pneumonia and include: persistent cough, shortness of breath, pyrexia, myalgia and fatigue. Other non-specific symptoms may also be present, such as headache, nasal congestion, sore throat, diarrhoea, nausea and vomiting [15].

The World Health Organization stipulates that the diagnosis of COVID-19 should be suspected in patients who present with acute respiratory illness in conjunction with a history of travel to a high prevalence area; or contact with a confirmed or suspected COVID-19 case within 14 days prior to symptoms onset. Additionally, acute respiratory illness in the absence of a more likely alternative diagnosis should prompt suspicion of COVID-19 [16]. In practice in the UK currently, suspicion of COVID-19 infection should be determined clinically, regardless of travel or contact history.

Certain groups, particularly the elderly and those with significant comorbidities such as cardiovascular disease, hypertension and diabetes are at increased risk of severe disease [17]. The severity of illness varies between cases; 80.9% of patients present with mild illness which does not require hospital admission. Conversely, 4.7% of cases require intensive care admission. The remainder, 13.8%, are severe cases that require hospital admission but not intensive care [17].

Clinical examination will reveal signs consistent with an upper or lower respiratory tract infection. The patient's observations may show pyrexia, tachypnoea, tachycardia and hypoxia. Examination of the chest may show inspiratory crackles, which is consistent with underlying consolidation.

NICE guidelines suggest that, at the time of initial presentation, an assessment of frailty using the Clinical Frailty Score (CFS) should be made and documented in the patient's notes [18]. The CFS considers age, functional baseline and comorbid conditions, grading patients from ‘1 – very fit’ to ‘9 – terminally ill.’ The score can assist clinicians in planning ceilings of care for COVID-19 patients.

Early discussion regarding suitability for intensive care admission are warranted, as well as consideration of patient preferences. Patients with a CFS score less than 5 (mildly frail) are most likely to benefit from organ support measures in critical care. Conversely, patients with a CFS score greater than 5 are less likely to benefit from such treatment. Early discussion regarding resuscitation is also warranted; patients with a CFS score greater than 5 are less likely to benefit [18]. Clinicians can supplement the CFS score with their own knowledge of the patient's comorbidities and severity of illness.

## 3 Investigations

Patients presented to the emergency department or acute medical take are likely to have basic investigations, such as blood tests and a chest x-ray, taken. In addition, diagnostic testing using a nasopharyngeal or oropharyngeal swab is now commonly completed. Furthermore, some centres conduct CT scans on patients presenting with the most severe symptoms.

Blood results in COVID-19 infection show derangements in multiple biomarkers. Isolated lymphopaenia has been noted in multiple studies with incidence ranging from 43 to 83% [14,20,21,45]. Elevations in acute phase reactants have been noted, with elevated c-reactive protein (CRP) and ferritin [19,26], consistent with systemic inflammatory response. Other common biomarker derangements noted in the literature include elevated lactate dehydrogenase (LDH), elevated liver enzymes (notably without derangements of liver synthetic function), and elevated D-dimer levels [[19], [20], [21],^26^]. These derangements have been studied for their utility as biomarker predictors of severity of COVID-19 infection. Evidence of correlation between severity and biomarker derangement has been demonstrated for lymphopaenia, CRP, LDH, liver enzymes, d-dimer, and ferritin [20,[27], [28], [29]]. Elevated d-dimer (>1000 ng/ml) was found to significantly predict in-hospital death, alongside age and SOFA score, with an odds ratio of 18.42 [30]. There is evidence that procalcitonin is commonly normal in patients and elevated levels may indicate secondary super-added bacterial infection, however the test is not widely available in the UK [31].

Diagnostic testing is being performed using real-time reverse transcriptase polymerase chain reaction (rRT-PCR) testing. Current PHE guidelines recommend an upper respiratory tract sample (combination of nose and throat swab) and a lower respiratory tract sample, if possible, to be sent for testing [32]. Comparison of specimen sites for COVID-19 has found positive rates in nasal swabs of 63%, with sputum and bronchoalveolar lavage having higher positive rates [33]. Analysis of a large cross-sectional study suggests a sensitivity of RT-PCR of 66–80% with 23% of initial negative tests becoming positive following repeat with 67% of these having initial CT images consistent with COVID-19 [34]. Repeat testing has been found to improve sensitivity from 71% on initial testing to 94% on second swab taken 1–2 days later [35]. Typical CT changes in patients with initially negative RT-PCR swabs have been reported, suggesting importance of repeat samples in those with CT changes consistent with COVID infection [35,36].

**Fig. 1:**
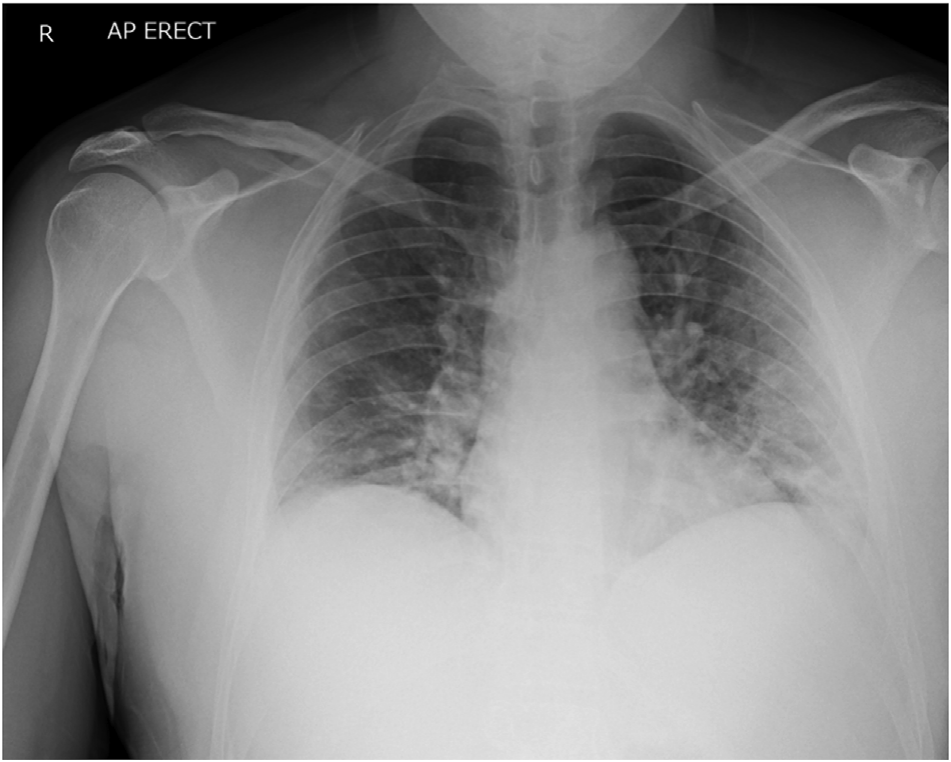
Chest radiograph from day of admission of 38 year-old male presenting with 7 day history of cough and fever. Radiograph shows bilateral infiltrates.

**Fig. 2:**
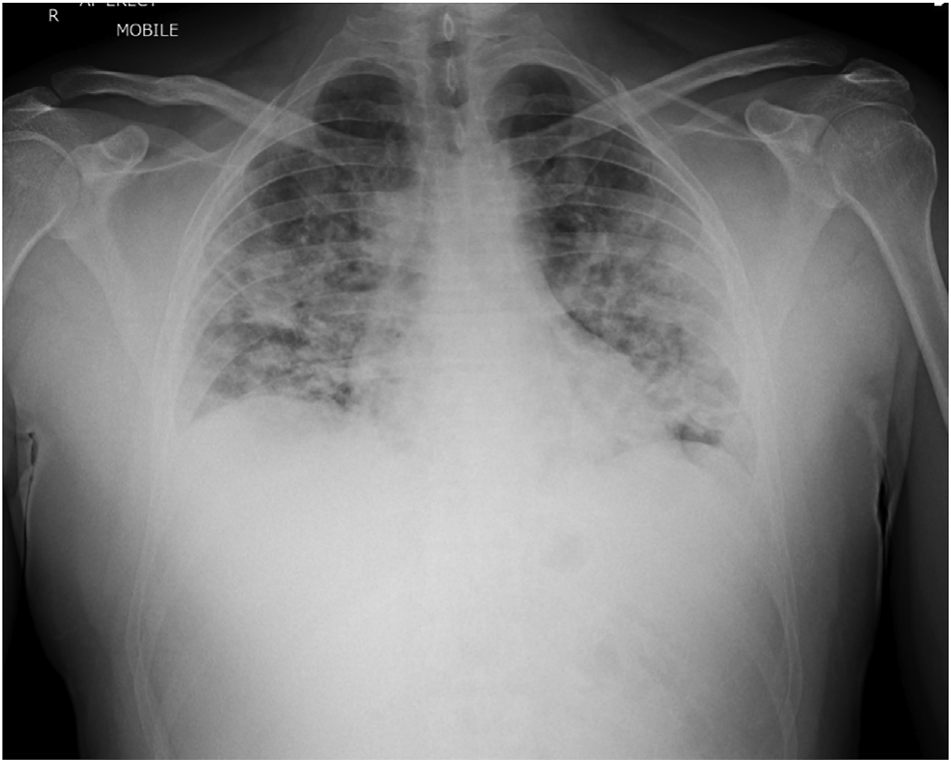
Chest radiograph of 38 year-old male, five days progressed from Fig. 1. Patient requiring increased oxygen supplementation and displaying increased work of breathing. Radiograph shows progression of bilateral infiltrates.

**Fig. 3:**
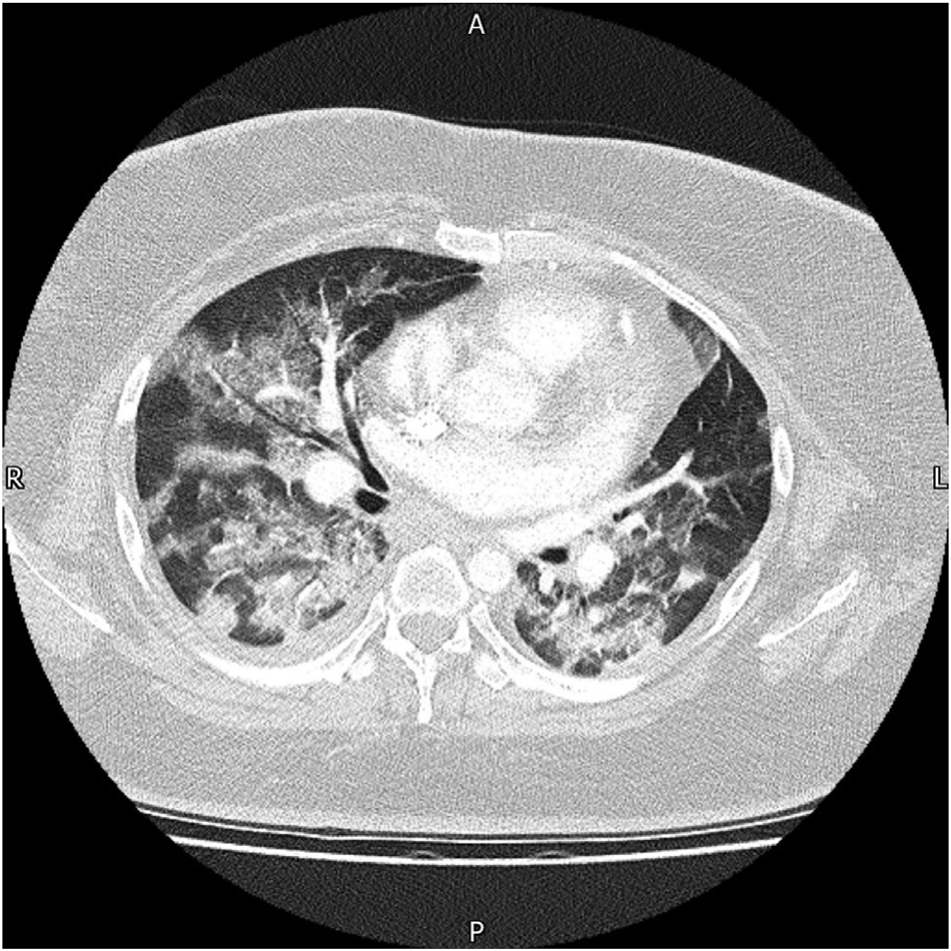
CT imaging of a 34-year-old female, who required intubation and ventilation for severe type 1 respiratory failure due to COVID-19. Bilateral ground glass opacification is demonstrated.

Radiological changes have been strongly associated with COVID-19 infection, with prevalence ranging from 86 to 100% [14,20,21]. A cohort of patients with asymptomatic and mild infection had abnormal CT findings in 57% of cases [37]. There is a relative paucity in the literature of simple radiograph findings as compared to CT. A recent study aiming to address this demonstrated chest x-ray to have a sensitivity of 69% with bilateral, lower zone consolidation and ground glass opacities predominating [38]. This fits with the authors anecdotal experience (Fig. 1, Fig. 2). In a large cross-sectional study, CXR changes (patchy or ground glass opacities) were noted in 59% of patients with the same cohort having CT abnormalities in 86% [21]. Commonly noted CT features include bilateral or multi-lobar changes, peripheral distribution, ground glass or patchy opacities, and consolidation [21,25,39,40]. Septal thickening, linear opacities, and air bronchograms have been described, with lymphadenopathy, pleural effusions, and cavitations notably absent as features of COVID-19 [39,[41], [42], [43]]. CT changes have been linked to severity with progression of ground-glass opacities and presence of consolidation being associated with more severe infection (Fig. 3) [[44], [45], [46], [47]].

## 4 Management

### 4.1 Initial management

Infection prevention and control in the form of personal protective equipment (PPE) and patient isolation are the most important initial steps in managing suspected COVID-19 infection [[16], [48]]. Public Health England recently published updated guidelines with regards to the use of PPE in secondary care setting [49].

Despite intensive research into targeted therapy, supportive measures currently form the basis of management. Supplemental oxygen should be titrated according to pulse oximetry and empirical antibiotics given to cover all likely pathogens of acute respiratory infection. Intravenous fluids should be given conservatively to avoid exacerbation of ARDS, of which noncardiogenic pulmonary oedema is the hallmark feature [16].

Hypoxemic respiratory failure secondary to ARDS is the most common cause of deterioration and death, with one report documenting 67% of COVID-19 ITU admissions developing ARDS [50]. Prompt recognition and escalation of severe COVID-19 disease allows early intervention. A trial of high-flow nasal oxygen may avoid the need for invasive endotracheal intubation [51]. ARDS lung protection ventilation guidelines are likely to minimise risk of ventilator-induced lung injury [52]. Prone positioning improves lung mechanics and has been shown to improve 90-day mortality in severe ARDS [53]. Fluid management and vasopressors are important in managing septic shock. Extra-corporeal membrane oxygenation is available at some centres and may be used as a last resort. A retrospective study of six COVID-19 patients treated with ECMO showed a mortality rate of 83.3% [50] Evidence suggests ECMO may be beneficial in severe ARDS [54].

### 4.2 Hydroxychloroquine and azithromycin

The safety of hydroxychloroquine, a drug used against malaria and autoimmune diseases, was established in the 1950s. The drug, on the WHO list of essential medicines, was recognised to have broad-spectrum anti-viral activity in 2006, and most recently was shown to be highly effective in controlling 2019-nCov infection [55,56]. Azithromycin, a macrolide antibiotic used commonly for a wide range of bacterial infections, has previously shown *in vitro* efficacy against zika and rhinoviruses [57,58].

A recent open-label non-randomised clinical trial in 42 patients comparing treatment with either hydroxychloroquine treatment or combination hydroxychloroquine-azithromycin treatment reported a significant reduction in viral carriage at day 6 post inclusion compare to standard of care. Combination treatment also showed significantly increased efficiency in viral elimination, with 100% of patients virologically cured at day 6, versus 12.5% in the control group (p < 0.001) [59]. Subsequent studies have disputed the methodology of the aforementioned study, throwing the validity of its results into question [60,61]. A recent systematic review in February identified twenty-three clinical trials of hydroxychloroquine or chloroquine pending approval or recruiting [62]. Notably, combination use with azithromycin is associated with QT prolongation and so caution must be taken in patients at risk of arrythmias due to comorbidity or polypharmacy [63].

### 4.3 Immunosuppression

There appears to be a subset of severe COVID-19 patients who develop hyperinflammation due to an underlying cytokine storm syndrome. Two studies showed an association between glucocorticoid therapy and clinical improvement and reduced inflammatory cytokine levels in SARS patients in the 2003 epidemic [64,65]. Two further studies however demonstrated an association between glucocorticoid therapy and increased 30-day mortality, and delayed viral clearance in SARS and MERS patients respectively [66,67]. Corticosteroids are currently not routinely recommended in COVID-19 due to potential exacerbation of lung injury. There are currently RCTs of intravenous methylprednisolone and Tocilizumab (an IL-6 receptor blocker licensed for cytokine storm syndrome) ongoing in COVID-19 patients [67,68].

### 4.4 Remdesivir

Remdesivir, a broad-spectrum anti-viral agent developed in 2017 as a treatment for Ebola virus, had its development halted when a randomised controlled trial of 673 participants with Ebola virus disease showed that 28-day mortality was significantly inferior in the Remdesivir group versus monoclonal antibodies mAb114 and REGN-EB3. There are currently eight RCTs registered specifically investigating the safety and efficacy of Remdesivir in treating COVID-19 patients [69]. No results are yet available.

### 4.5 Lopinavir/ritonavir

Lopinavir/ritonavir, a combination antiretroviral medication used to treat human immunodeficiency virus (HIV), has been studied for the treatment of COVID-19. A randomized-controlled trial of 199 hospitalized participants showed no benefit of lopinavir/ritonavir in reducing time to clinical improvement, mortality and serum viral load beyond standard care [70].

### 4.6 NSAIDs

Concerns regarding use of NSAIDs in COVID-19 have been raised since French Health Minister Olivier Véran released a statement advising against the use of ibuprofen in COVID-19 due to potential worsening of severity of symptoms [71]. NHS England circulated an email from the NHS Medical Director Professor Stephen Powis on March 17, 2020 stating that the Commission of Human Medicines and NICE have been asked to review the current evidence and formulate formal guidance, but in the interim the advice is to use paracetamol to treat fever in COVID-19, avoiding the use of NSAIDs [72]. It stated that patients established on NSAID treatment for other reasons should not discontinue use. The WHO will also gather further evidence before making a formal recommendation.

## 5 Conclusion

The COVID-19 pandemic is evolving, with numbers infected and deaths attributed to the disease increasing rapidly. This has led to a global effort to create widely available detection assays and to identify a targeted treatment. The cornerstone of management throughout the world is currently supportive management with oxygen therapy alongside treatment for sepsis and ARDS with broad-spectrum antibiotics and careful rationing of intravenous fluids. Stratification of risk is a priority, with a focus on identifying those most at need and most likely to benefit from invasive ventilation. Though some facilities are trialling specific treatments, with hydroxychloroquine and azithromycin most commonly used among these, there are no widely used guidelines as to their use as yet. There is a plethora of expedited clinical trials currently ongoing [73].

## Ethical approval

Nil required.

## Sources of funding

Nil.

## Author contribution

Billy Down – conceptualisation, writing original draft, refinement and editing.

Sagar Kulkarni - conceptualisation, writing original draft, refinement and editing.

Ameer Hamid Ahmed Khan - writing original draft, refinement and editing.

Benjamin Barker - writing original draft, refinement and editing.

Ivan Tang - refinement and editing.

## Registration of research studies

1 Name of the registry:
2 Unique Identifying number or registration ID:
3 Hyperlink to your specific registration (must be publicly accessible and will be checked):

## Guarantor

Billy Down.

## Provenance and peer review

Not commissioned, externally peer reviewed.

## Declaration of competing interest

The authors of this article have no conflicts of interest to declare.
